# Supplementary material for: A probabilistic map of emotional experiences during competitive social interactions
Source: Nat Commun. 2022 Mar 31;13:1718. doi: 10.1038/s41467-022-29372-8 (PMC8971394; doi:10.1038/s41467-022-29372-8)
Supplement: Supplementary file 1 — Supplementary Information [file 41467_2022_29372_MOESM1_ESM.pdf]

Supplementary information

for

A probabilistic map of emotional experiences during competitive social interactions

Table of Contents

Supplementary Discussion **2**

1 Emotion Classification Task 2

2 Experiment 1: Ultimatum Game 7

2.1 Responders and Third Parties Respond Similarly 7

2.2 Affective Experiences Predict Punishment 7

2.3 Neural Network Results 13

2.4 k-means Clustering Results 14

2.5 kNN Classification Results 15

2.6 Euclidean Distance Analysis 16

3 Experiment 2: Prisoner's Dilemma 18

3.1 Discrete Contribution Analysis 18

3.2 Continuous Contribution Analysis 19

3.3 Conditional Contribution Analysis 21

3.4 Euclidean Distance Analysis 22

4 Experiment 3: Public Goods Game 24

4.1 Discrete Contribution Analysis 24

4.2 Continuous Contribution Analysis 25

4.3 Conditional Contribution Analysis 26

4.4 Euclidean Distance Analysis 27

Supplementary References **29**

Supplementary Discussion

1 Emotion Classification Task

The variability associated with affective ratings from the emotion classification task can be visualized in two-dimensions or one-dimension, depending on the researcher's goal. We visualized the two-dimensional density of each emotion using contour lines in the manuscript but here visualize density using color, similar to a heatmap (Supplementary Figure 1). Because the absolute level of density is not the same across all emotions, the color mapping is the relative density within a specific emotion category. It is likely that the highest density regions reflect the canonical emotion response associated with each category, but these also reveal nuances which may be meaningful to specific emotion categories depending on theoretical points. These differences can be meaningfully quantified, as visualized in one-dimensional density plots (Supplementary Figure 2). For example, comparing variability of across affective dimensions might be important for emotions thought to be relatively homogenous. We compared the standard deviations of valence and arousal ratings between all emotions using a F-test and visualized the Bonferroni corrected significant results (Supplementary Figure 3). Another way to analyze these distributions is to see whether participants may rely on different interpretations of the emotion word (e.g., relaxed), which would result in a multimodal distribution (e.g., two modes at the qualitatively different interpretations). Researchers have used several measures to distinguish between unimodality and multimodality, but one particularly robust metric is known as the Hartigan's dip statistic (HDS; Freeman & Dale, 2013; Hartigan & Hartigan, 1985)). For example, in the case of the valence ratings for the emotion word "relaxed", results show that the distribution is significantly different from a unimodal distribution (dip statistic D = 0.0650, $p < .001$). If we assume that this distribution is bimodal, then one possible explanation for the distribution is that participants are reflecting on two distinct experiences of the emotion "relaxed" – some participants experienced this emotion as being neither pleasant nor unpleasant (neutral) while others rated this experience as highly pleasant. Supplementary Tables 1 and 2 contain uncorrected HDS tests for all emotions except neutral (because neutral is bound according to the exclusion criteria) on both the arousal and valence dimensions. These analyzes

help reveal the structure of emotions on the affective dimensions and demonstrate the heterogeneity of emotional experiences.

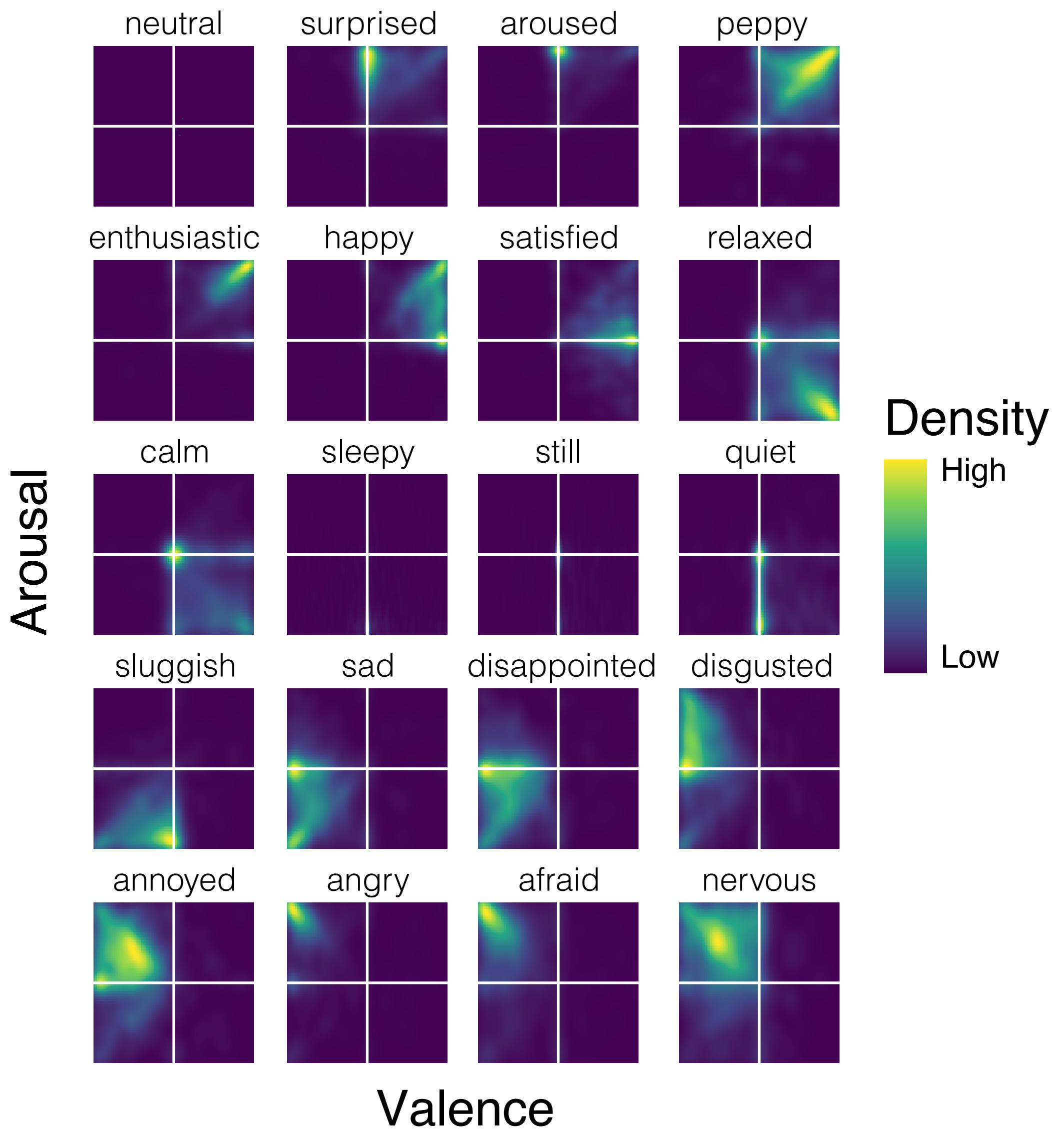

Supplementary Figure 1. 2D Density Plots from the Emotion Classification Task. *The x-axis represents the valence dimension, the y-axis represents the arousal dimension, and color represents the relative density of valence-arousal ratings (yellow = high density, blue = low density). Density levels are relative to each emotion class.*

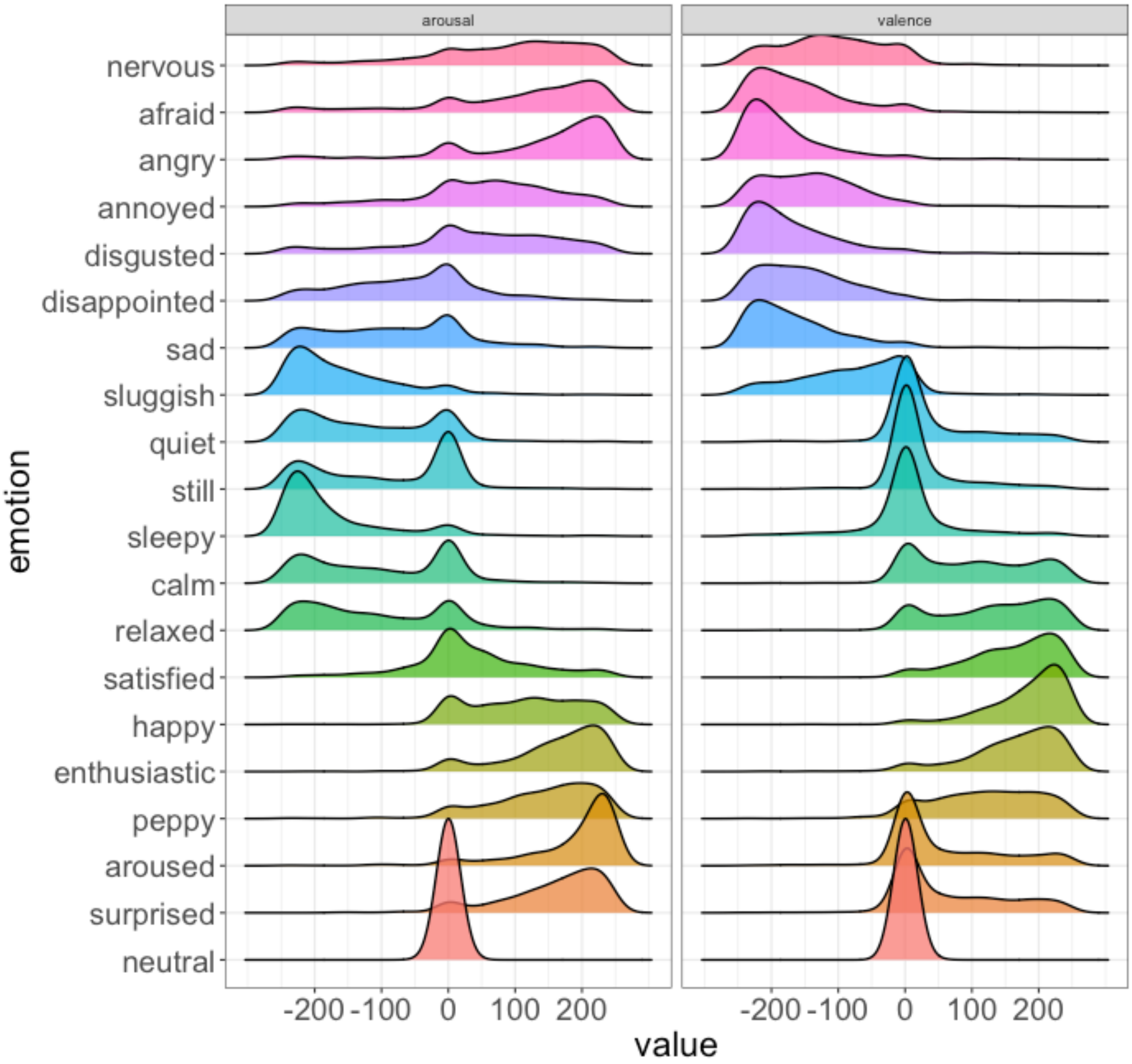

Supplementary Figure 2. 1D Density Plots from the Emotion Classification Task. *The x-axis represents the affect rating on either the valence dimension or the arousal dimension. The y-axis represents the density of the affect ratings, which vary for each emotion class and affect measurement. Density plots were generated with a scale of 3 and a bandwidth of 18.*

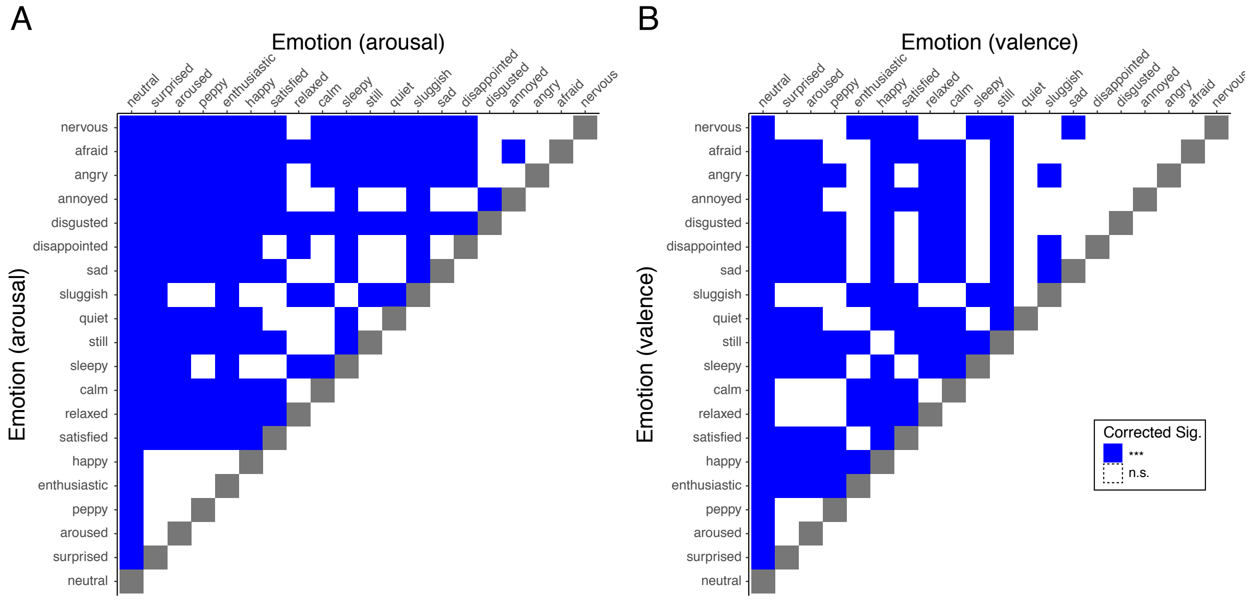

Supplementary Figure 3. Variance comparisons with F-tests. *Pairwise variance ratio tests were performed for all emotions on the arousal (A) and valence (B) dimensions. Blue tiles indicate significant variance differences after Bonferroni correction with arousal and valence comparisons.*

**Emotion Label**	**Valence Distribution Type**	**Valence HDS**
Afraid	Multimodal	0.015*
Angry	Unimodal	0.01
Annoyed	Multimodal	0.014*
Aroused	Multimodal	0.052***
Calm	Multimodal	0.026***
Disappointed	Unimodal	0.008
Disgusted	Unimodal	0.009
Enthusiastic	Multimodal	0.015*
Happy	Unimodal	0.012
Nervous	Multimodal	0.03***
Peppy	Multimodal	0.026***
Quiet	Multimodal	0.056***
Relaxed	Multimodal	0.055***
Sad	Unimodal	0.01
Satisfied	Unimodal	0.012
Sleepy	Multimodal	0.054***
Sluggish	Multimodal	0.02***
Still	Multimodal	0.07***
Surprised	Multimodal	0.042***

Supplementary Table 1. Multimodality in valence experiences by emotion categories. *Evidence for a multimodal distribution was determined through Hartigan's dip statistic (HDS). HDS is a null-hypothesis test, therefore a significant result indicates the rejection of a unimodal*

distribution. The distribution column indicates whether the valence distribution is unimodal or multimodal according to the HDS. All p-values are uncorrected for multiple comparisons.

Emotion Label	Arousal Distribution Type	Arousal HDS
Afraid	Multimodal	0.028***
Angry	Multimodal	0.039***
Annoyed	Multimodal	0.018**
Aroused	Unimodal	0.013
Calm	Multimodal	0.061***
Disappointed	Multimodal	0.015*
Disgusted	Multimodal	0.015*
Enthusiastic	Multimodal	0.025***
Happy	Multimodal	0.036***
Nervous	Multimodal	0.024***
Neutral	Multimodal	0.098***
Peppy	Multimodal	0.018**
Quiet	Multimodal	0.066***
Relaxed	Multimodal	0.065***
Sad	Multimodal	0.016**
Satisfied	Multimodal	0.024***
Sleepy	Multimodal	0.022***
Sluggish	Multimodal	0.016**
Still	Multimodal	0.067***
Surprised	Multimodal	0.02***

Supplementary Table 2. Multimodality in arousal experiences by emotion categories. *Evidence for a multimodal distribution was determined through Hartigan's dip statistic (HDS). HDS is a null-hypothesis test, therefore a significant result indicates the rejection of a unimodal distribution. The distribution column indicates whether the arousal distribution is unimodal or multimodal according to the HDS. All p-values are uncorrected for multiple comparisons.*

2 Experiment 1: Ultimatum Game

2.1 Responders and Third Parties Respond Similarly

Participants either played the UG as the Responder or a non-vested third party. Using a logistic mixed-effects model, we examined whether affective reactions to offers depended on the participants' role. Results showed a main effect of role, such that third parties punished more than Responders, but no significant interaction between valence and role or arousal and role (Supplementary Table 3). Thus, despite the difference in their roles, all participants responded similarly on valence and arousal to unfair offers.

Responders and Third Parties Respond Similarly

	Estimates			
Predictors	*Log-Odds*	*std. Error*	*Statistic*	*p*
Intercept	-3.80	0.34	-11.12	**<0.001**
Valence	-5.34	0.36	-14.80	**<0.001**
Arousal	-0.06	0.41	-0.16	0.874
Role [Self]	-1.22	0.45	-2.74	**0.006**
Valence:Arousal	-0.41	0.34	-1.23	0.220
Valence:Role [Self]	0.38	0.45	0.85	0.396
Arousal:Role [Self]	0.32	0.46	0.68	0.495
Valence:Arousal:Role [Self]	0.32	0.36	0.89	0.373
N_{sub}	364			

Supplementary Table 3. Responders and Third Parties Respond Similarly*. Valence and arousal have been scaled, but not mean-centered, as the 0 point refers to the meaningful instance when affect is neutral. Role is operationalized as self or responder (0) and third party (1). The mixed-effects model included a random intercept and random slopes for valence, arousal, and their interaction per subject and conducts two-sided t-tests for each coefficient without correction for multiple comparison.*

2.2 Affective Experiences Predict Punishment

Decisions in the Ultimatum Game (UG) may involve a policy decision (e.g., if offer below 20% of the total, reject) and here we test whether affective experiences explain decisions to punish

above and beyond such a policy decision. We used mixed-effects logistic regressions to test two models of decision-making in the UG: one which models choices to punish as a function of unfairness (Supplementary Equation 1), and one which models choices to punish as a function of unfairness interacting with affective experiences (valence and arousal separately). Supplementary Equation 1 captures the intuition that people use a policy decision (e.g., reject if below 20% of the total pie) as the slope of unfairness (β_1) will represent the strength of the policy decision while the intercept (β_0) will represent the point of indifference where participants are 50% likely to punish. (vs accept). Supplementary Equation 2 captures the intuition that emotional affective experiences improve predicting decisions to punish and potentially adjust sensitivity to unfairness.

$$punish \sim \beta_0 + \beta_1 unfairness \quad (1)$$

$$punish \sim \beta_0 + \beta_1 valence + \beta_2 arousal + \beta_3 unfairness + \beta_4 valence * unfairness + \beta_5 arousal * unfairness \quad (2)$$

We used likelihood ratio tests to compare these nested models and infer which model better explains decisions to punish. Likelihood ratio tests reveal that the model which includes affective experiences (Supplementary Equation 2) significantly improves the fit compared to the model which only included unfairness ($\chi^2(11) = 489.21,\ p < .001$). Results from Supplementary Equation 2 show significant marginal effects of unfairness and valence such that higher unfairness increases the probability of rejection while more positive valence decreases the probability (Supplementary Table 4). In short, a simpler model based purely on a decision policy of amount of money offered does a worse job of explaining behavior compared to a model that includes affective experiences.

Ultimatum Game Affect Model of Punishment

	**Estimates**			
Predictors	*Log-Odds*	*std. Error*	*Statistic*	*p*
Intercept	-4.71	0.28	-16.61	**<0.001**
Unfairness	3.85	0.26	14.95	**<0.001**
Valence	-2.28	0.22	-10.31	**<0.001**

Arousal	-0.00	0.18	-0.02	0.982
Unfairness:Valence	-0.13	0.15	-0.91	0.363
Unfairness:Arousal	0.19	0.13	1.48	0.138
Valence:Arousal	-0.09	0.14	-0.62	0.535
Unfairness:Valence:Arousal	0.20	0.11	1.88	0.060
N_{sub}	715			

Supplementary Table 4. Ultimatum Game Affect Model of Punishment. *Valence and arousal have been scaled, but not mean-centered, as the 0 point refers to the meaningful instance when affect is neutral. Unfairness is operationalized as the amount of money kept by Player B and has been normalized (scaled and mean-centered). The mixed-effects model included a random intercept and random slopes for unfairness, valence, and arousal per subject and conducts two-sided t-tests for each coefficient without correction for multiple comparison.*

We also examined how valence and arousal combine to predict decisions to punish. Using a mixed-effect regression, we tested whether the interaction between valence and arousal predicts decisions to punish using the following fixed-effects formulation (Supplementary Equation 3):

$$punish \sim \beta_0 + \beta_1 valence + \beta_2 arousal + \beta_3 valence * arousal \qquad 3$$

Results from this mixed-effect regression show that while arousal on its own does not predict decisions to punish, there is a significant interaction between valence and arousal (Supplementary Table 5). This suggests that the joint combination of valence and arousal are critical in understanding how emotions relate to punishment. Given the variability in arousal ratings associated with decisions to punish, we also tested the possibility that arousal might predict decisions to punish in a non-linear way. We added a quadratic term for arousal to Supplementary Equation 3 and results show a significant non-linear effect of arousal, such that increases in the quadratic value of arousal strongly predicts punishment (Supplementary Table 6). Supplementary Figure 4 visualizes the main effect of arousal in this non-linear model, illustrating how the probability of punishing increases at the two extremes of the arousal scale. Furthermore, the visualized interaction between valence and arousal (Supplementary Figure 5) reveals how the relationship between valence and punishment is significantly attenuated when

arousal is neutral (arousal = 0 SD) compared to when arousal is extremely low (arousal = -2 SD) or extremely high (arousal = +2 SD).

Interaction between Valence and Arousal Predicts Punishment

	**Estimates**			
Predictors	*Log-Odds*	*std. Error*	*Statistic*	*p*
Intercept	-3.88	0.18	-21.64	**<0.001**
Valence	-4.51	0.18	-25.51	**<0.001**
Arousal	-0.23	0.19	-1.22	0.222
Valence:Arousal	-0.38	0.16	-2.42	**0.015**
N_{sub}	715			

Supplementary Table 5. Interaction Between Valence and Arousal Predicts Punishment. *Valence and arousal have been scaled, but not mean-centered, as the 0 point refers to the meaningful instance when affect is neutral. The mixed-effects model included a random intercept and random slopes for valence, arousal, and their interaction per subject and conducts two-sided t-tests for each coefficient without correction for multiple comparison.*

Non-linear Effects of Arousal Predicting Punishment

	**Estimates**			
Predictors	*Log-Odds*	*std. Error*	*Statistic*	*p*
Intercept	-3.43	0.16	-20.89	**<0.001**
Valence	-4.20	0.17	-24.16	**<0.001**
Arousal(1)	7.33	11.91	0.62	0.538
Arousal(2)	66.66	10.05	6.63	**<0.001**
Valence:Arousal(1)	-24.75	9.36	-2.64	**0.008**
Valence:Arousal(2)	-12.80	8.92	-1.44	0.151
N_{sub}	715			

Supplementary Table 6. Non-linear Effects of Arousal Predicting Punishment. *Valence and arousal have been scaled, but not mean-centered, as the 0 point refers to the meaningful instance when affect is neutral. Orthogonal polynomials were used to test for non-linear effects of arousal and the degree of the polynomial is presented in paratheses (e.g., (1), (2)). The mixed-effects model included a random intercept and random slopes for valence and the orthogonal*

polynomial terms for arousal per subject and conducts two-sided t-tests for each coefficient without correction for multiple comparison.

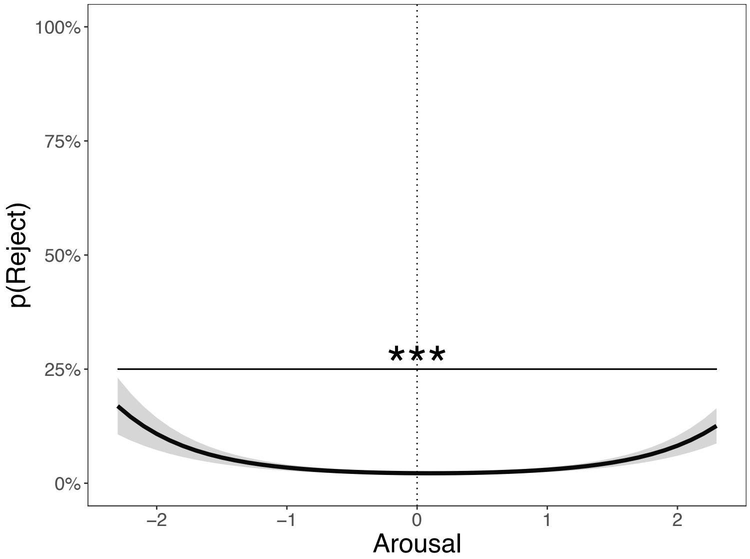

Supplementary Figure 4. Main Effect of Arousal from Supplementary Table 6. *The predicted regression line for the main effect of arousal from Table 3 is plotted for continuous values of arousal when valence equals 0. Valence and arousal were scaled, but not mean-centered, as the 0 point meaningfully refers to the case when affect is neutral. Error bars are +/- 1 standard errors of the mean.*

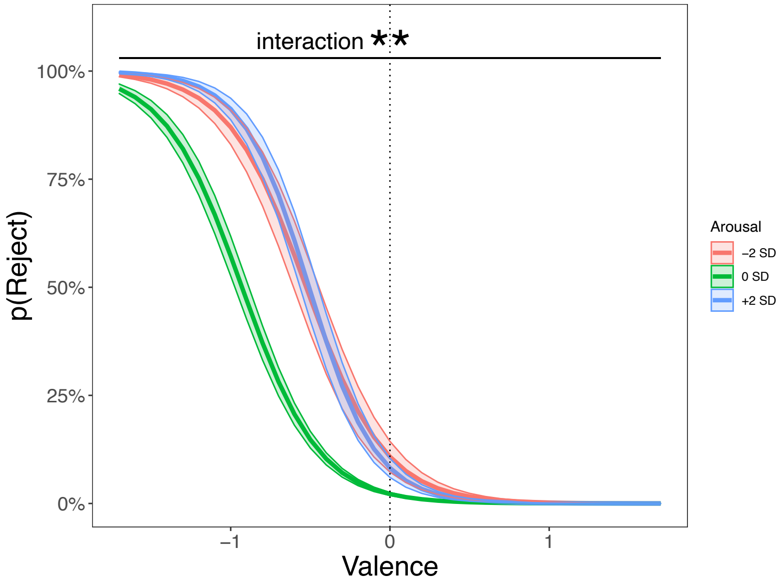

Supplementary Figure 5. Interaction Between Valence and Arousal from Supplementary Table 6. *Predicted regression lines from Table 3 are plotted for continuous values of valence and binned values of arousal. For visualization purposes arousal has been binned to be -2, 0, or +2 standard deviation, although the regression used the continuous value. Valence and arousal*

were scaled, but not mean-centered, as the 0 point meaningfully refers to the case when affect is neutral. Error bars are +/- 1 standard errors of the mean.

2.3 Neural Network Results

We applied the trained neural network to the affective experiences in the Ultimatum Game to generate model likelihoods for each emotion category for decisions to punish and accept. While the information from this analysis is displayed in the manuscript (Figure 4), we visualized it a different way that highlights the conditional probabilities of each choice given a specific emotion (i.e., *p(punish | emotion)*; Supplementary Figure 6). Using two-sample t-tests, we compared the model likelihoods associated with punishment decisions and accept decisions for each emotion (going from left to right emotions in Supplementary Figure 6). Results from this analysis show that punishment decisions are significantly more likely than accept decisions when the emotion is sadness, disappointed, disgust, annoyed, anger, afraid, nervous, or sluggish (all P's < .001). Conversely, accept decisions are significantly more likely than punish decisions when the emotion is still, quiet, calm, peppy, neutral, relaxed, satisfied, surprised, aroused, enthusiastic, or happy (all P's < .001). For the emotion sleepy, accept is significantly more likely than punish, although the effect is much weaker than the other emotions (t(942.97) = 2.021, p = .04).

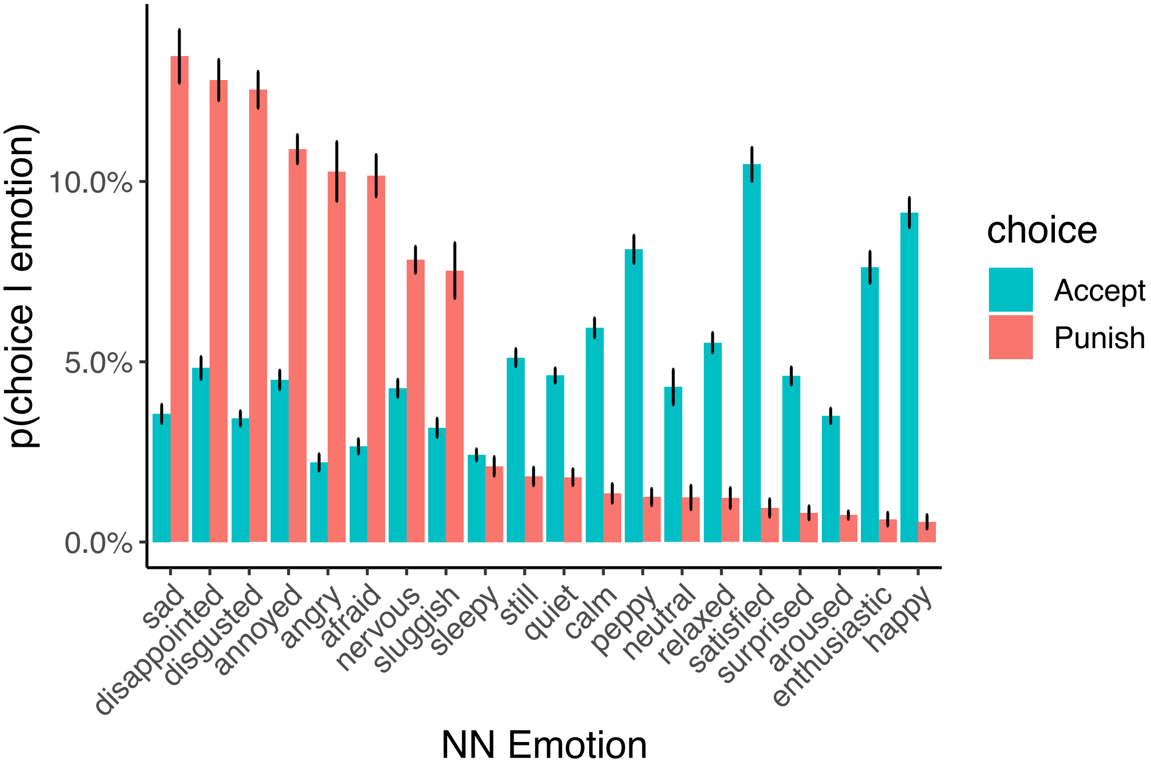

Supplementary Figure 6. Conditional probabilities of choice by neural network emotion classifications. *The neural network model trained on the emotion classification data was applied to the unlabeled affect ratings from the Ultimatum Game. Each data point was assigned a probability of each emotion class and these were averaged within, then across participants (N = 715), and across choices. Emotions are organized by the conditional probability of choice given emotion for each emotion class. Data are presented as mean values while errors bars reflect 95% CIs.*

2.4 k-means Clustering Results

We used a k-means clustering algorithm to classify affective experiences in the Ultimatum Game into one of nine distinct clusters representing low, medium, and high valence and arousal areas. We analyzed and visualized the proportion of punish decisions within each cluster and the proportion of each decision per cluster (Supplementary Figure 7). It is important to distinguish the sensitivity of a cluster to a particular choice (Supplementary Figure 7A) from the representation of that cluster inside of all decisions to punish or accept (Supplementary Figure 7B). For example, Clusters 1 and 3 contain a high percentage of punishment decisions within each cluster respectively (Cluster 1: 70.6%, Cluster 3: 58.2%; Figure S6A). However, Cluster 1 and 3 only represent 3.4% and 11.5%, respectively, of all decisions to punish (Figure S6B). In other words, although Cluster 1 is comprised of mostly punishment decisions, the base rate of punishment decisions falling into Cluster is very low.

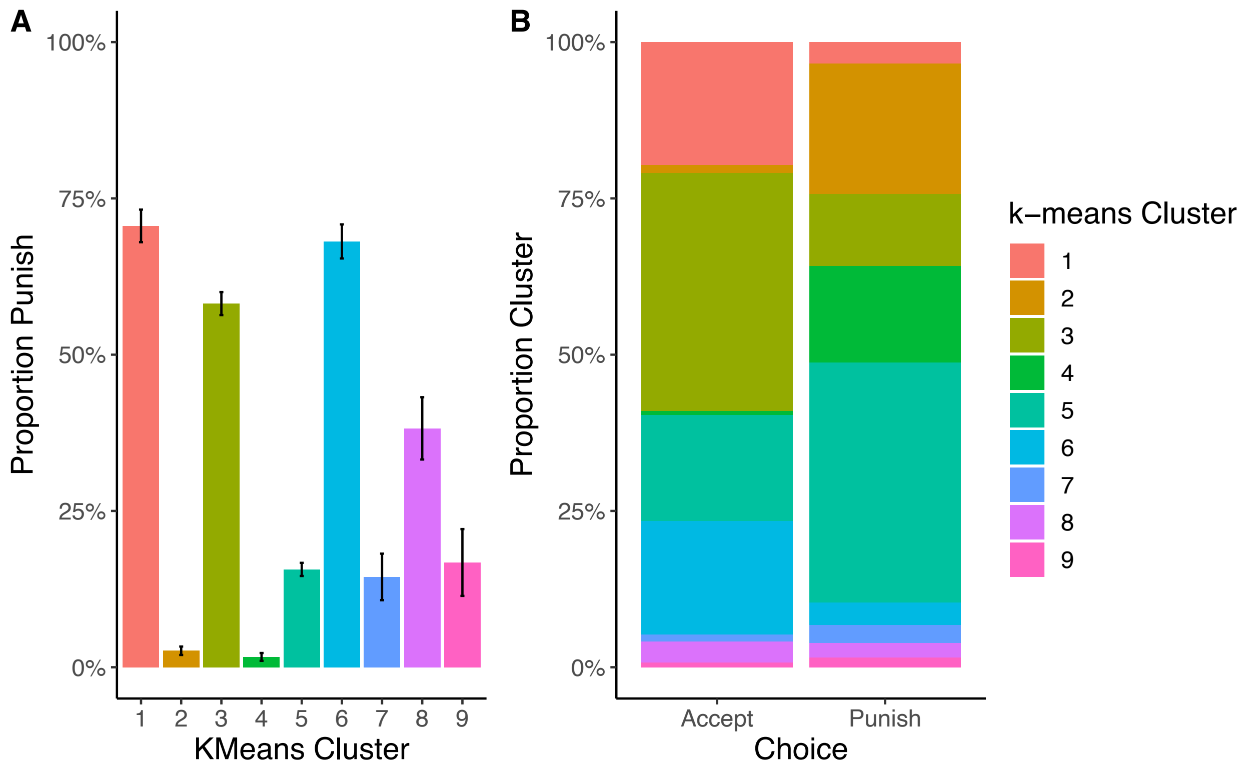

Supplementary Figure 7. k-means clustering proportions. A) Proportion of punish decisions by clusters. *Affective experiences in the Ultimatum Game were clustered into 9 clusters using k-means clustering. Bars represent the proportion of punishment decisions averaged within participants (N = 715) and then within each cluster. Data are presented as mean values while error bars reflect 95% CIs.* ***B) Proportion of clusters by choices****. Stacked bars represent the proportion of clusters which fall into accept or punish decisions.*

2.5 kNN Classification Results

Although we used the neural network algorithm as the final classification model in the manuscript, the kNN classification algorithm accuracy was very similar to the neural network. Here, we examine the average model likelihoods associated with each emotion class and decisions to punish using the kNN algorithm. Results show that the ranking of emotions is virtually identical to the neural network results presented in the manuscript (Supplementary Figure 8).

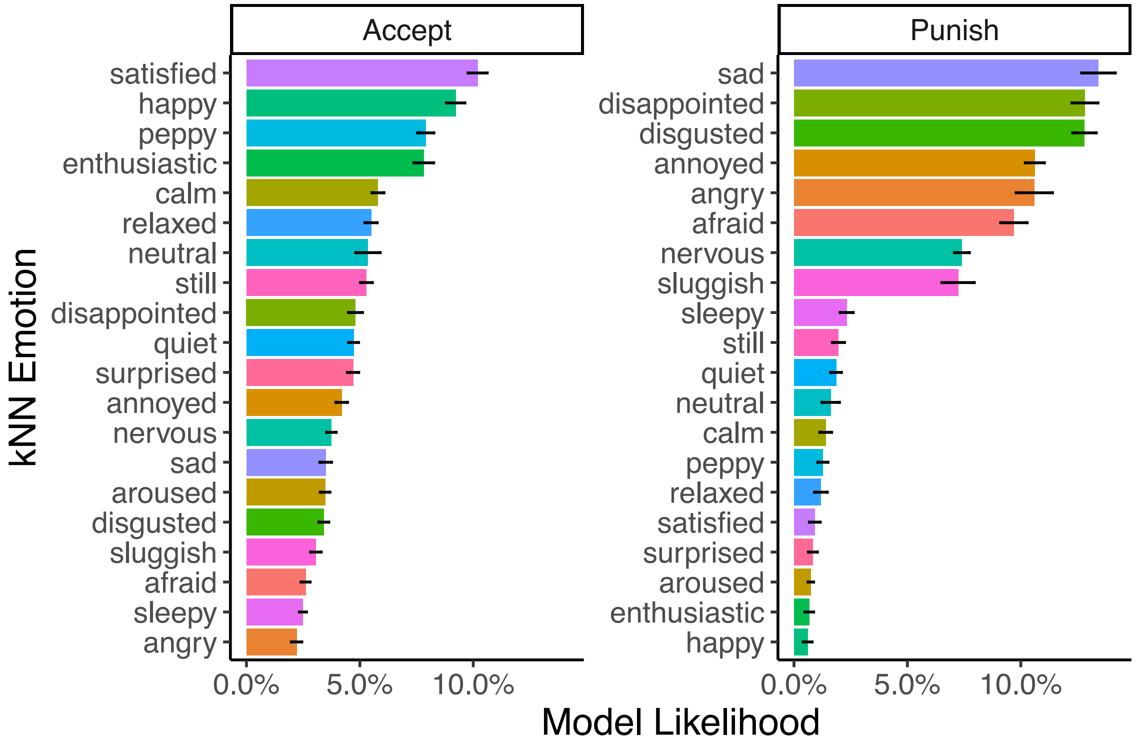

Supplementary Figure 8. k-nearest neighbors emotion classifications by decisions to accept and punish*. The k-nearest neighbors model trained on the emotion classification data was applied to the unlabeled affect ratings from the Ultimatum Game. Each data point was assigned a probability of each emotion class and model likelihoods were averaged within participant (N = 715) and then across choice. Data are presented as mean values while error bars reflect 95% CIs.*

2.6 Euclidean Distance Analysis

We also developed an emotion classifier that is unique to each participant by calculating the Euclidean distance between all 20 emotion labels and the affective experience during each trial of the UG. We convert Euclidean distance into a probability using inverse distance weighting, where probability is the inverse of the Euclidean distance for a specific emotion over the sum of inverse Euclidean distances of all emotions. This way smaller Euclidean distances indicate a higher probability that the affective experience matched the valence and arousal rating of the emotion class for each participant separately. Results show that disgust, disappointment, and anger are the top three emotions associated with punishing (Supplementary Figure 9).

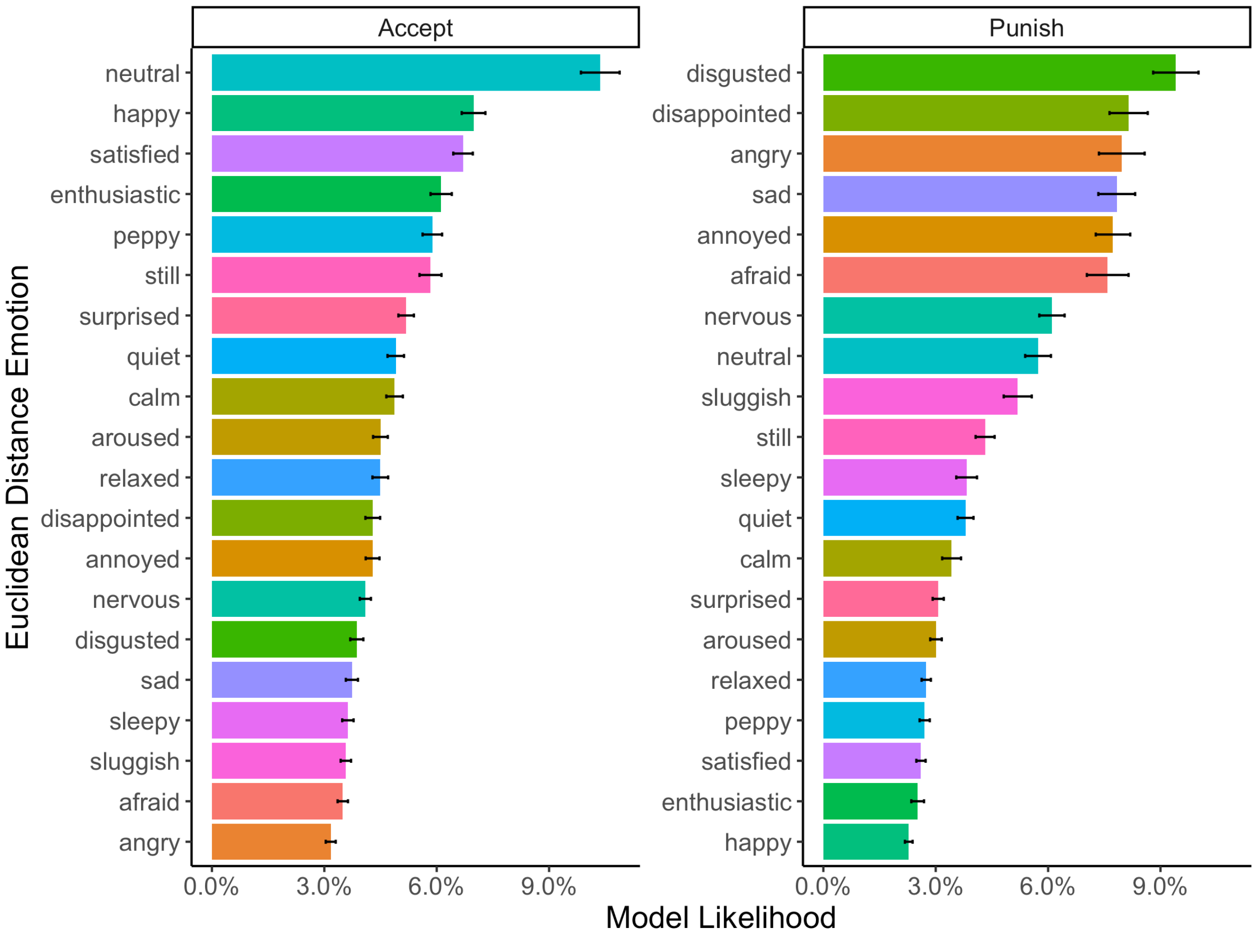

Supplementary Figure 9. Euclidean distance classifier for the UG*. Inverse distance weighting was used to calculate the probability of each emotion class for all unlabeled emotion ratings in the Ultimatum Game. This classification was done separately for each participant (N = 715), and model likelihoods were averaged within participant and then across participants. Data are presented as mean values while error bars reflect 95% CIs.*

3 Experiment 2: Prisoner's Dilemma

3.1 Discrete Contribution Analysis

We binned continuous contributions ($0 - $1) made in the Prisoner's Dilemma (PD) into decisions to defect ($0 - $0.49) and cooperate ($0.50 - $1), and classified emotions experienced in the PD using the trained neural network from the emotion classification task. Results reveal that the top three emotions associated with decisions to defect are disappointment (8.83%), sadness (8.72%), and disgust (7.38%), with anger (5.11%) being identified as the 9th most likely emotion to be experienced in the Prisoner's Dilemma (Supplementary Figure 10). Paired t-tests revealed that disappointment (t(278) = 8.26, $p < .001$, $d = .50$), sadness ($t(278) = 6.84$, $p < .001$, $d = .41$), and disgust ($t(278) = 8.37$, $p < .001$, $d = .50$) were all significantly more likely to be associated with punishment than anger. When cooperating, the top three emotions most likely to be experienced were happiness, satisfaction, and enthusiasm.

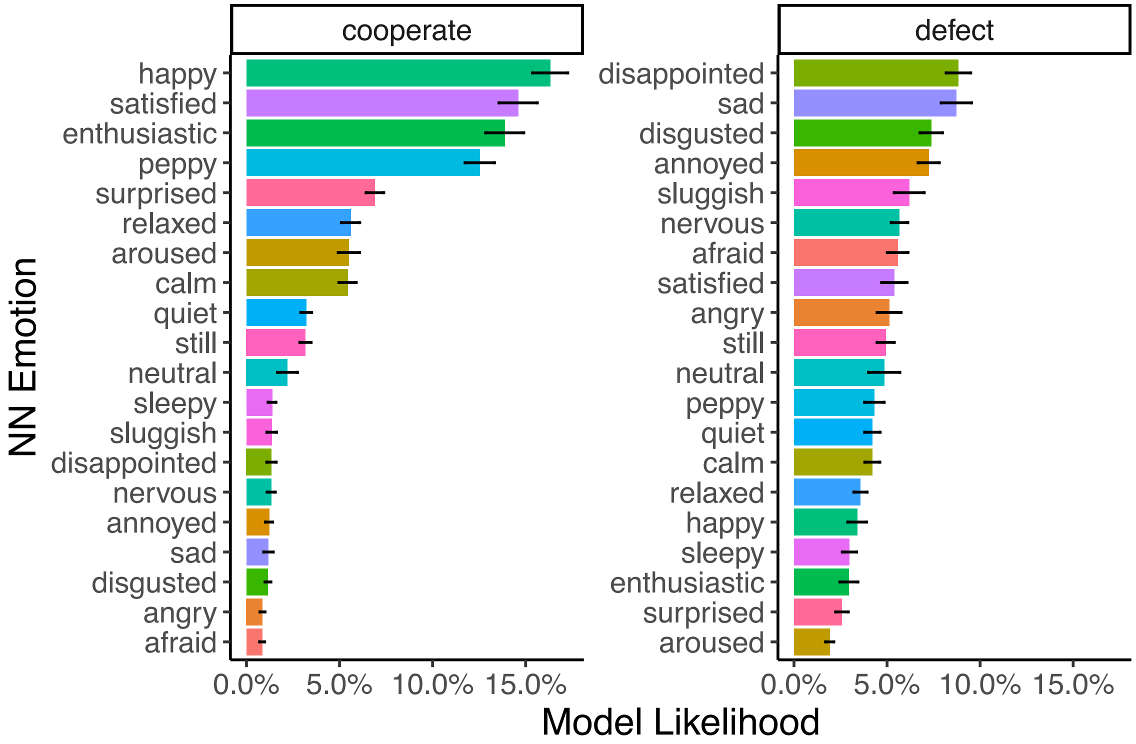

Suppelementary Figure 10. Classifications by decisions to cooperate and defect. *The neural network model trained on the emotion classification data was applied to the unlabeled emotion ratings from the Prisoner's Dilemma. Each data point was assigned a probability of each emotion*

class, and model likelihoods were averaged within participant (N = 306) and then across participants. Data are presented as mean values while error bars reflect 95% CIs.

3.2 Continuous Contribution Analysis

We investigated how the probability of any specific emotion classification changed if we model continuous contributions ($0 - $1) rather than discrete contributions. We applied the trained neural network reported in the manuscript on the unlabeled emotion experiences in the PD which results in a classification probability for each of the twenty emotion terms. We ran separate mixed-effects linear regressions for each emotion term, using the participant's contribution to predict the neural network probability for that emotion term (Supplementary Figure 11). This analysis allows for more granular changes as participants increase their contributions in the PD. Examining the slopes of these regressions indexes the strength of the relationship between contributions in the PD and the probability of a specific emotion being experienced (Supplementary Figure 12). Stronger slopes indicate a stronger relationship between that emotion and contributions in the PD. For example, if monetary contributions in the PD are tightly coupled with feelings of anger, this analysis should demonstrate a strong slope between contributions and the probability of the emotion being classified as anger. Results show that compared to other emotions (i.e., sad, disappointed, and disgusted), anger was not as sensitive to changes in contribution levels. This demonstrates that even when taking the continuous nature of the contributions into account, anger is not a representative emotion associated with defection.

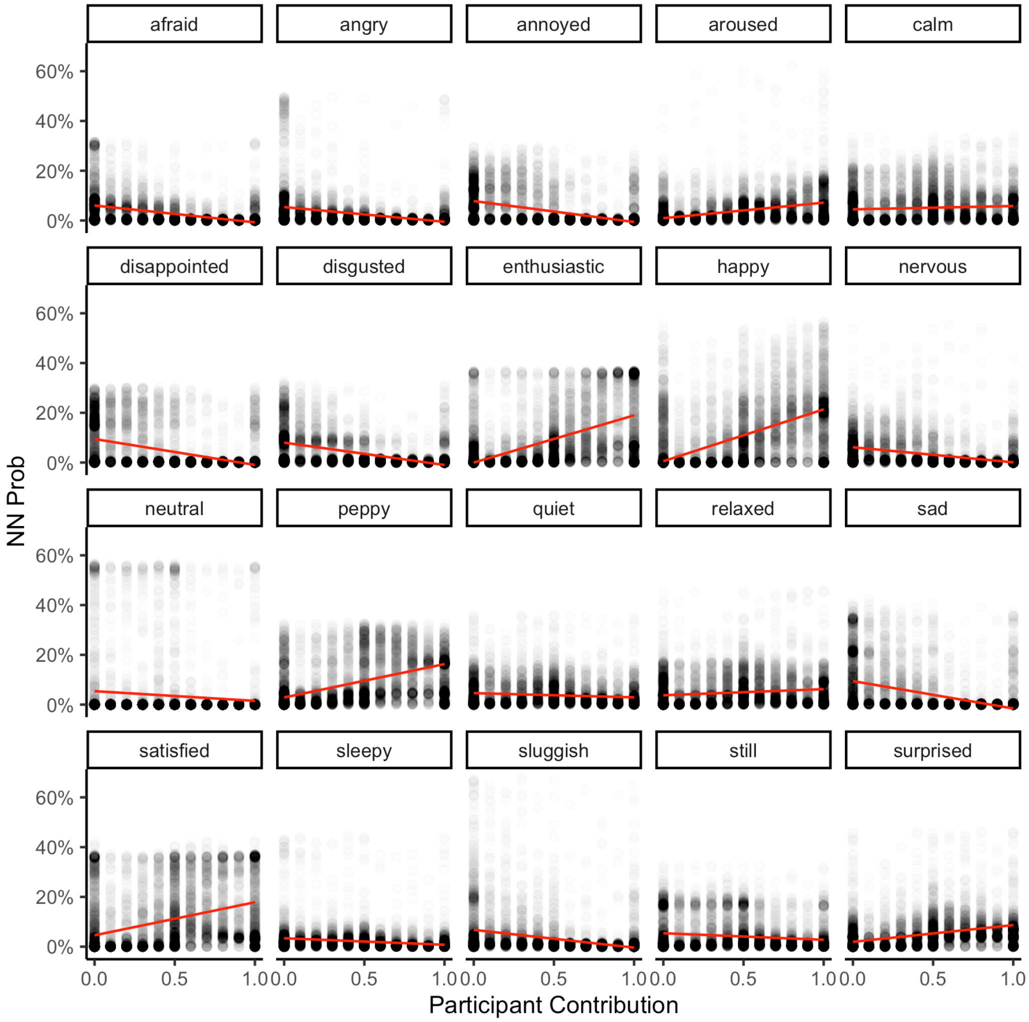

Supplementary Figure 11. Classifications by continuous contributions in the PD. *The neural network model trained on the emotion classification data was applied to the unlabeled emotion ratings from the Prisoner's Dilemma. Each data point was assigned a probability of each emotion class and is associated with a specific continuous contribution. Red lines reflect the fixed-effect predictions from separate mixed-effects regressions for each emotion class.*

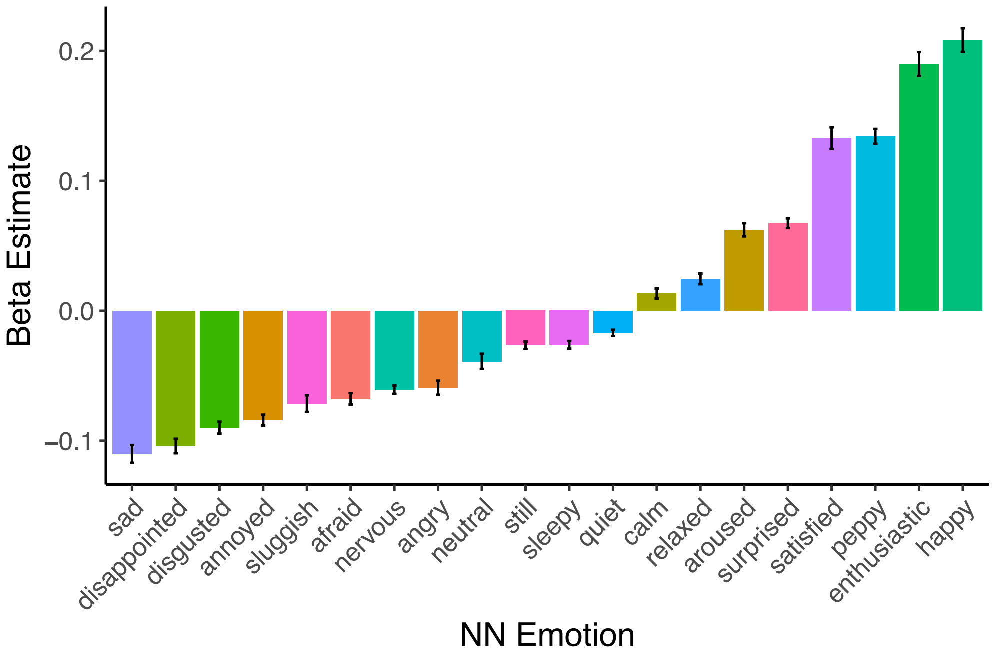

Supplementary Figure 12. Relationship between contributions and emotion class probability. *Separate mixed-effects regressions were run for each emotion class predicting the probability of that emotion class as a function of contributions in the PD for participants (N = 306). Bars reflect the fixed-effect slope of contributions for each emotion class. Higher estimates indicate that increasing contributions increased the probability of this emotion class. Data are presented as beta coefficients while error bars reflect standard errors.*

3.3 Conditional Contribution Analysis

We also investigated how the probability of any specific emotion classification changed if we model conditional cooperation. Participants played a sequential Prisoner's Dilemma, meaning that participants contributions were made after their partners and therefore represent conditional cooperation. Because the relationship between conditional cooperation and neural network emotion probabilities is largely non-linear, we used separate loess regressions for each emotion class (Supplementary Figure 13). Results show that emotions have different relationships with conditional cooperation – for example, participants are very likely to feel happy when completely defecting against their partners (conditional contribution = -1) while emotions such as angry are most high when conditional cooperation is high (conditional contribution = +1). Although participants most often matched their partner's contribution, one explanation for these puzzling results is that some participants consistently contributed nothing \$0 or everything \$1, effectively

deciding before seeing their partner's contribution, and these relationships may reflect post-decision emotions rather than pre-decision emotions.

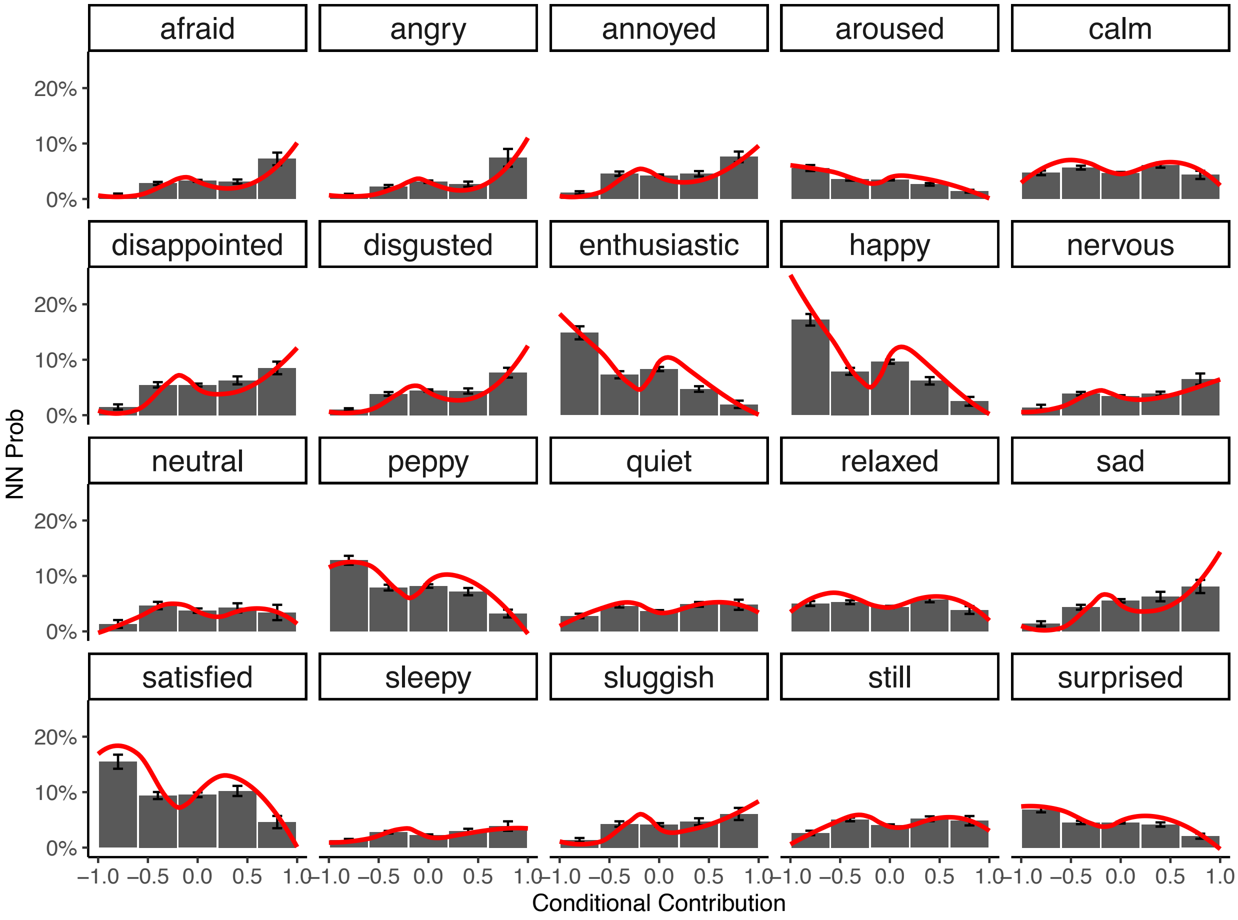

Supplementary Figure 13. NN Probabilities by Conditional Cooperation in the PD. *Conditional cooperation is the difference between the participant's contribution and their partner. Conditional cooperation has been binned into five bars to visualize the non-linear relationship between conditional cooperation and neural network probabilities. Data are presented as mean values while error bars reflect 95% CIs. Red lines are loess regression lines using the continuous conditional cooperation measure for each emotion category for participants (N = 306).*

3.4 Euclidean Distance Analysis

We also developed an emotion classifier that is unique to each participant by calculating the Euclidean distance between all 20 emotion labels and the affective experience during each trial of the PD. Results show that neutral, disappointment, and disgust are the top three emotions associated with defecting (Supplementary Figure 14).

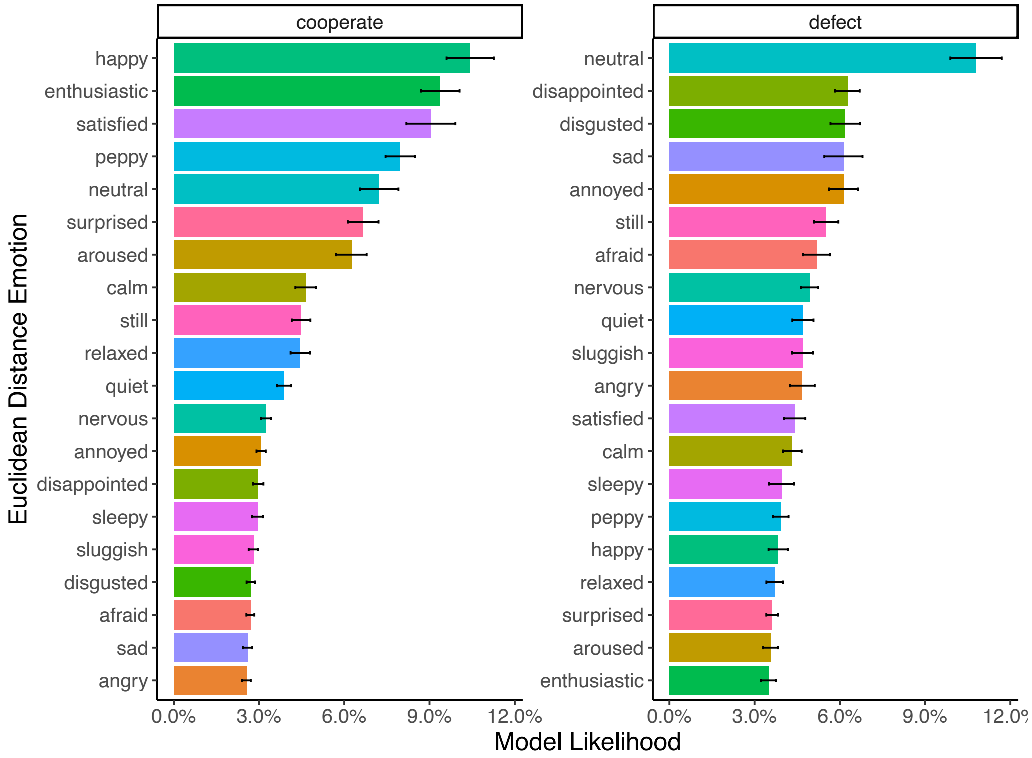

Supplementary Figure 14. Euclidean distance classifier for the PD*. Inverse distance weighting was used to calculate the probability of each emotion class for all unlabeled emotion ratings in the Prisoner's Dilemma. This classification was done separately for each participant (N = 306), and model likelihoods were averaged within participant and then across participants. Data are presented as mean values while error bars reflect 95% CIs.*

4 Experiment 3: Public Goods Game

4.1 Discrete Contribution Analysis

We binned continuous contributions (\$0 - \$1) made in the Prisoner's Dilemma (PD) into decisions to defect (\$0 - \$0.49) and cooperate (\$0.50 - \$1), and classified emotions experienced in the PD using the trained neural network from the emotion classification task. Results reveal that the top three emotions associated with decisions to defect are sadness (10.36%), disappointment (9.63%), and sluggishness (8.38%), with anger (4.74%) being identified as the 8th most likely emotion to be experienced in the Public Goods Game (Supplementary Figure 15). Paired t-tests revealed that sadness (t(443) = 12.9, $p < .001$, $d = .61$), disappointment ($t(443) = 14.0$, $p < .001$, $d = .67$), and sluggishness ($t(443) = 7.60$, $p < .001$, $d = .36$) were all significantly more likely to be associated with punishment than anger. When cooperating, the top three emotions most likely to be experienced were happiness, enthusiasm, and satisfaction.

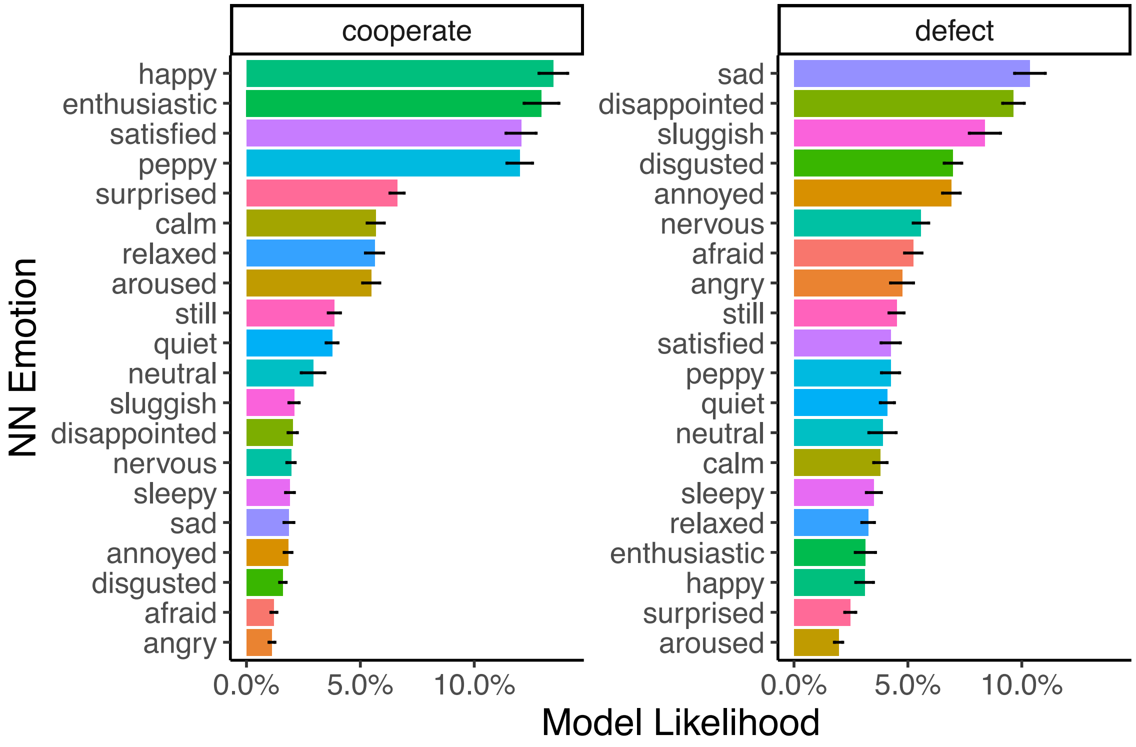

Supplementary Figure 15. Classifications by decisions to cooperate and defect in the PGG. *The neural network model trained on the emotion classification data was applied to the unlabeled*

emotion ratings from the Public Goods Game. Each data point was assigned a probability of each emotion class and model likelihoods were averaged within participant (N = 470) and then across participants. Data are presented as mean values while error bars reflect 95% CIs.

4.2 Continuous Contribution Analysis

We again investigated how the probability of any specific emotion classification might change with continuous contributions ($0 - $1) in the PGG. We used separate mixed-effects regressions to examine the relationship between emotion probabilities and participant contributions. Results revealed that sadness, disappointment, and sluggishness are more sensitive to changes in a participant's contributions, when compared to anger (Supplementary Figure 16; Supplementary Figure 17).

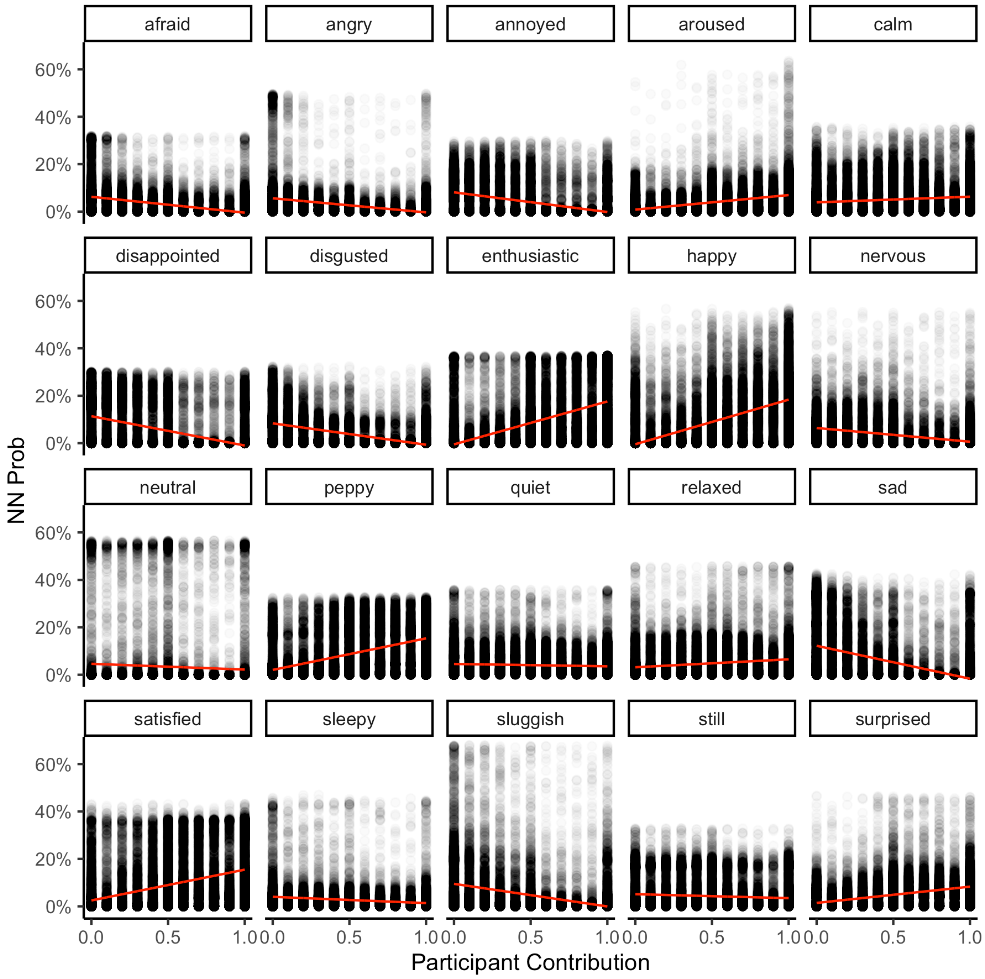

Supplementary Figure 16. Classifications by continuous contributions in the PGG. *The neural network model trained on the emotion classification data was applied to the unlabeled emotion*

ratings from the Public Goods Game. Each data point was assigned a probability of each emotion class and is associated with a specific continuous contribution. Red lines reflect the fixed-effect predictions from separate mixed-effects regressions for each emotion class.

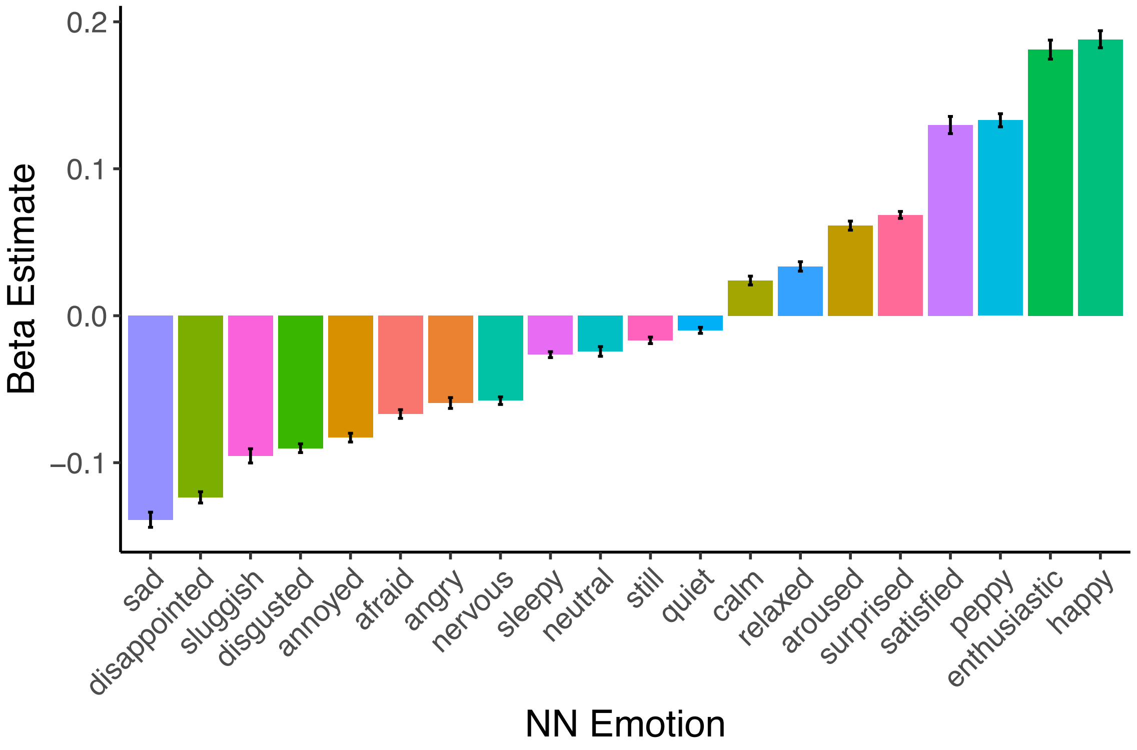

Supplementary Figure 17. Relationship between contributions and emotion class probability. *Separate mixed-effects regressions were run for each emotion class predicting the probability of that emotion class as a function of contributions in the PGG for participants (N = 470). Bars reflect the fixed-effect slope of contributions for each emotion class. Higher estimates indicate that increasing contributions increased the probability of this emotion class. Data are presented as beta coefficients while error bars reflect standard errors.*

4.3 Conditional Contribution Analysis

We also investigated how the probability of any specific emotion classification changed if we model conditional cooperation. Participants played a sequential Public Goods Game with three partners, meaning that participants contributions were made after their partners and therefore represent conditional cooperation. Participants were only shown the aggregate total of their partner's contribution, so we calculated conditional cooperation as the participant's contribution minus the average partner contribution (i.e., total contribution / 3). Because the relationship

between conditional cooperation and neural network emotion probabilities is largely non-linear, we used separate loess regressions for each emotion class (Supplementary Figure 18).

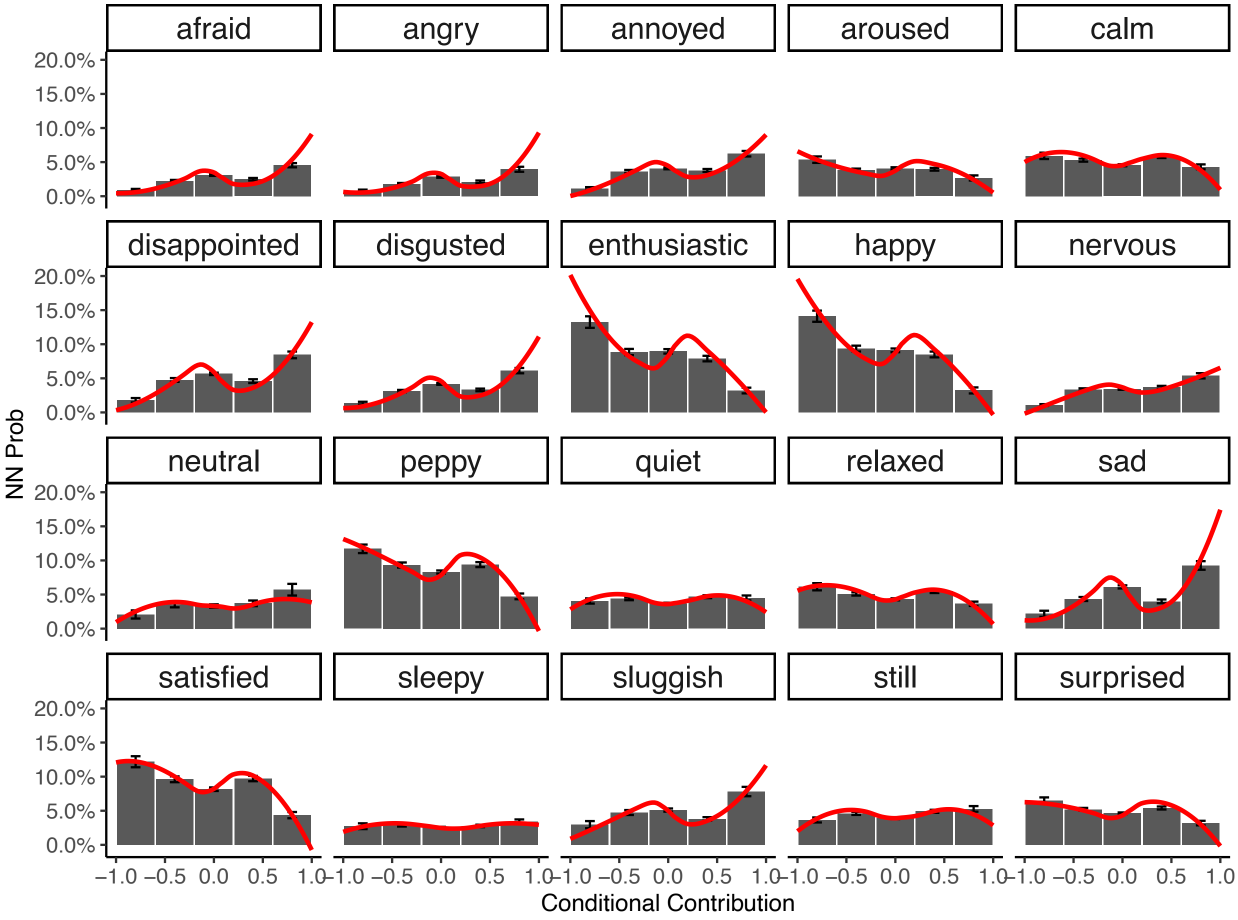

Supplementary Figure 18. NN Probabilities by Conditional Cooperation in the PGG. *Conditional cooperation is the difference between the participant's contribution and the average partner's contribution (total contribution / 3). Conditional cooperation has been binned into five bars to visualize the non-linear relationship between conditional cooperation and neural network probabilities. Data are presented as mean values while error bars reflect 95% CIs. Red lines are loess regression lines using the continuous conditional cooperation measure for each emotion category for participants (N = 470).*

4.4 Euclidean Distance Analysis

We also developed an emotion classifier that is unique to each participant by calculating the Euclidean distance between all 20 emotion labels and the affective experience during each trial of

the PGG. Results show that neutral, disappointment, and sadness are the top three emotions associated with defecting (Supplementary Figure 19).

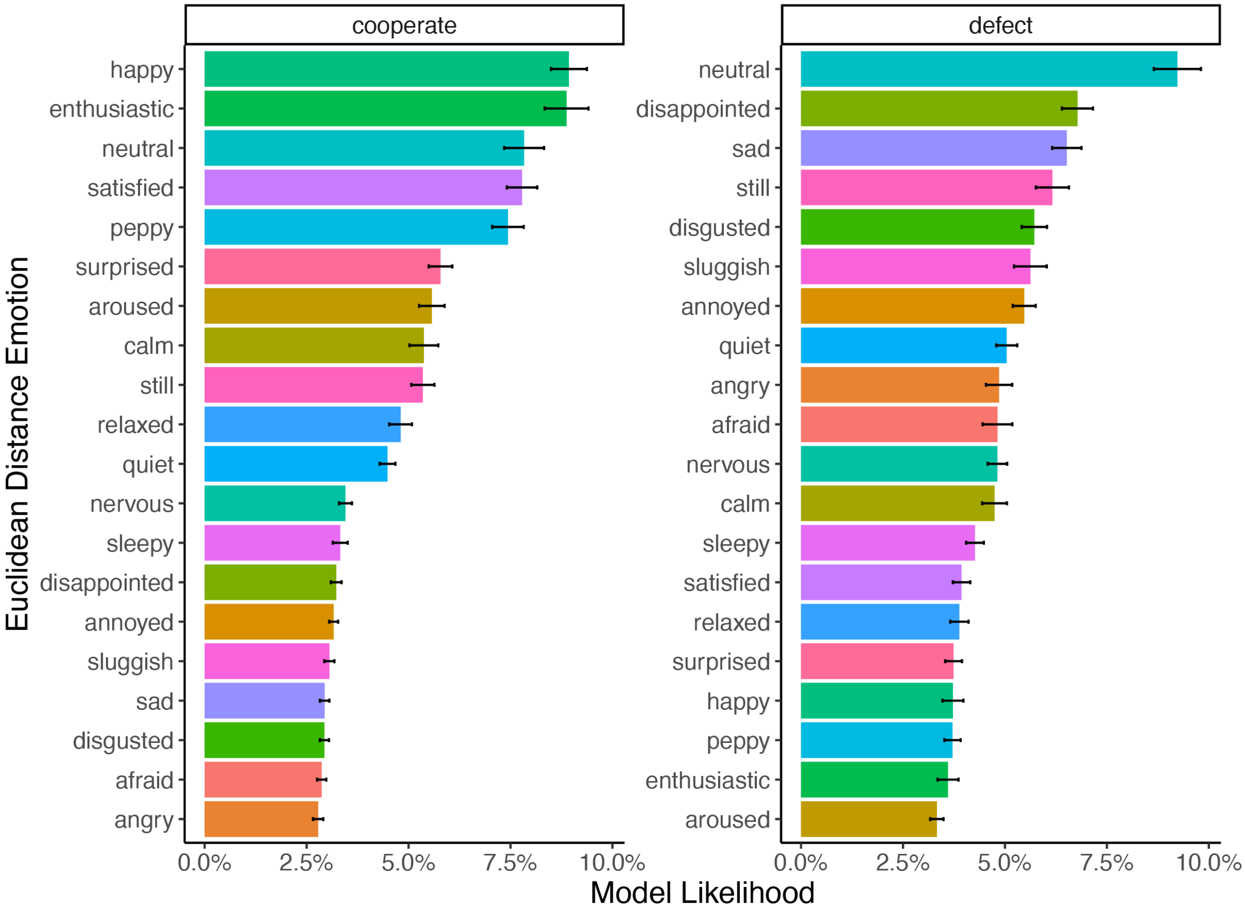

Supplementary Figure 19. Euclidean distance classifier for the PGG*. Inverse distance weighting was used to calculate the probability of each emotion class for all unlabeled emotion ratings in the Public Goods Game. This classification was done separately for each participant (N = 470), and model likelihoods were averaged within participant and then across participants. Data are presented as mean values while error bars reflect 95% CIs.*

Supplementary References

Freeman, J. B., & Dale, R. (2013). Assessing bimodality to detect the presence of a dual cognitive process. *Behav Res Methods, 45*(1), 83-97. doi:10.3758/s13428-012-0225-x

Hartigan, J. A., & Hartigan, P. M. (1985). The Dip Test of Unimodality. *The Annals of Statistics, 13*(1), 70-84. doi:10.1214/aos/1176346577
