# Supplementary material for: A probabilistic map of emotional experiences during competitive social interactions
Source: Nat Commun. 2022 Mar 31;13:1718. doi: 10.1038/s41467-022-29372-8 (PMC8971394; doi:10.1038/s41467-022-29372-8)
Supplement: Supplementary file 3 — Reporting summary [file 41467_2022_29372_MOESM3_ESM.pdf]

nature research

Corresponding author(s): Oriel FeldmanHall

Last updated by author(s): Dec 3, 2021

Reporting Summary

Nature Research wishes to improve the reproducibility of the work that we publish. This form provides structure for consistency and transparency in reporting. For further information on Nature Research policies, see our Editorial Policies and the Editorial Policy Checklist.

Statistics

For all statistical analyses, confirm that the following items are present in the figure legend, table legend, main text, or Methods section.

n/a	Confirmed	
☐	☒	The exact sample size (*n*) for each experimental group/condition, given as a discrete number and unit of measurement
☐	☒	A statement on whether measurements were taken from distinct samples or whether the same sample was measured repeatedly
☐	☒	The statistical test(s) used AND whether they are one- or two-sided<br>*Only common tests should be described solely by name; describe more complex techniques in the Methods section.*
☐	☒	A description of all covariates tested
☐	☒	A description of any assumptions or corrections, such as tests of normality and adjustment for multiple comparisons
☐	☒	A full description of the statistical parameters including central tendency (e.g. means) or other basic estimates (e.g. regression coefficient) AND variation (e.g. standard deviation) or associated estimates of uncertainty (e.g. confidence intervals)
☐	☒	For null hypothesis testing, the test statistic (e.g. F, t, r) with confidence intervals, effect sizes, degrees of freedom and P value noted<br>*Give P values as exact values whenever suitable.*
☒	☐	For Bayesian analysis, information on the choice of priors and Markov chain Monte Carlo settings
☐	☒	For hierarchical and complex designs, identification of the appropriate level for tests and full reporting of outcomes
☐	☒	Estimates of effect sizes (e.g. Cohen's d, Pearson's r), indicating how they were calculated

Our web collection on statistics for biologists contains articles on many of the points above.

Software and code

Policy information about availability of computer code

Data collection	Data for all experiments were collected using web-based surveys implemented on Mechanical Turk.
Data analysis	All data analysis code are available on the manuscript's associated Github https://github.com/jpheffne/NC_emotion_classify. Analyses were completed in R version 4.1.1.

For manuscripts utilizing custom algorithms or software that are central to the research but not yet described in published literature, software must be made available to editors and reviewers. We strongly encourage code deposition in a community repository (e.g. GitHub). See the Nature Research guidelines for submitting code & software for further information.

Data

Policy information about availability of data

All manuscripts must include a data availability statement. This statement should provide the following information, where applicable:

- Accession codes, unique identifiers, or web links for publicly available datasets
- A list of figures that have associated raw data
- A description of any restrictions on data availability

Experimental materials information and all experiment de-identified data are publicly available at https://github.com/jpheffne/NC_emotion_classify.
The materials used in this study are widely available.

Field-specific reporting

Please select the one below that is the best fit for your research. If you are not sure, read the appropriate sections before making your selection.

- [ ] Life sciences
- [x] Behavioural & social sciences
- [ ] Ecological, evolutionary & environmental sciences

For a reference copy of the document with all sections, see nature.com/documents/nr-reporting-summary-flat.pdf

Behavioural & social sciences study design

All studies must disclose on these points even when the disclosure is negative.

Study description	All experiments are experimental and contain an emotion classification task (rating 20 emotion words on valence and arousal dimensions) and a social exchange task involving monetary choices between participants and anonymous partners.
Research sample	The research samples for all studies consisted of workers from Amazon's Mechanical Turk. While not a fully representative sample, the participants are more diverse in age, race, and socioeconomic status than typical undergraduate research samples (Buhrmester, Kwang, & Gosling, 2011; Perspectives on Psychological Science). The final sample for Experiment 1 (UG) was N = 715 (320 Females, mean age = 34.4 +/- 10.1), the final sample for Experiment 2 (PD) was N = 306 (131 Females, mean age = 35.5 +/- 11.2), and the final sample for Experiment 3 (PGG) was N = 470 (238 Females, mean age = 33.0 +/- 10.5). The data from Experiment 1 comes from a larger dataset, portions of which have been previously published in Heffner, Son, & FeldmanHall, 2021.
Sampling strategy	All experiments used random sampling. In experiment 1, we planned to exceed sample sizes of past studies examining the relationship between emotions and punishment in the Ultimatum Game; in particular, Pillutla & Murnighan, 1989 found moderate correlations between anger and unfairness ratings with N = 118. Accordingly, we aimed to collect 700 participants to vastly exceed this sample size. We collected 906 Mechanical Turk participants and 191 participants were excluded for not following instructions, ending in a final sample of 715. The data from Experiment 1 comes from a larger dataset, portions of which have been previously published in Heffner, Son, & FeldmanHall, 2021. In experiment 2, we used a different experimental task and aimed to collect roughly 200 participants, based on our preregistration (https://osf.io/ypse2) and which exceeds sample sizes of past studies using the same economic game. We collected 395 Mechanical Turk participants and 89 participants were excluded for not following instructions, ending with a sample size of 306. In experiment 3, we aimed to collect 450. Using the same exclusion criterion, we collected 519 participants and excluded 49 due to noncompliance resulting in a final sample of 470.
Data collection	All data were collected online and used computer-based experiments, which measured participants responses and response times.
Timing	Data for Experiment 1 was collected between 8-28-2017 and 04-24-2019. Experiment 1 included 3 sample cohorts, with the first collected between 08-28-2017 and 08-30-2017; the second between 10-25-2017 and 10-30-2017; the third between 04-23-2019 and 04-24-2019.<br><br>Data for Experiment 2 was collected between 08-02-2019 and 08-14-2019. Data for Experiment 3 was collected between 08-21-2019 and 08-26-2019.
Data exclusions	In all 3 experiments participants were excluded based on our noncompliance policy described in our preregistration report (https://osf.io/ypse2) and prior studies (Heffner, Son, & FeldmanHall, 2021). This conservative measure of noncompliance required participants to correctly rate the 'neutral' feeling in the emotion classification task, which we explicitly instructed participants to rate in the center of a 500 x 500 pixel square (dARM). If participants neutral rating fell outside of a 100 x 100 square around the center, then they were excluded.
Non-participation	No participants dropped out or declined participation.
Randomization	In experiment 1, participants completed the Ultimatum Game either as Player B or Player C and allocation was random. There was little effect of role (Player B or C) on participant's emotional experiences. As such, experiments 2 and 3 had no between subject conditions.

Reporting for specific materials, systems and methods

We require information from authors about some types of materials, experimental systems and methods used in many studies. Here, indicate whether each material, system or method listed is relevant to your study. If you are not sure if a list item applies to your research, read the appropriate section before selecting a response.

Materials & experimental systems

n/a	Involved in the study
☒	☐ Antibodies
☒	☐ Eukaryotic cell lines
☒	☐ Palaeontology and archaeology
☒	☐ Animals and other organisms
☐	☒ Human research participants
☒	☐ Clinical data
☒	☐ Dual use research of concern

Methods

n/a	Involved in the study
☒	☐ ChIP-seq
☒	☐ Flow cytometry
☒	☐ MRI-based neuroimaging

Human research participants

Policy information about studies involving human research participants

Population characteristics	Experiment 1: n = 715 (320 female; mean age = 34.4; SD = 10.1). CES-D Center for Epidemiologic Studies Depression) scores were collected for 351 participants and 146 were at risk of depression while 205 were considered healthy controls.<br>Experiment 2: n = 306 (131 female; mean age = 35.5; SD = 11.2). CES-D scores were collected for all 306 participants and 132 were at risk of depression while 174 were considered healthy controls.<br>Experiment 3 n = 470 (238 female; mean age = 33.0; SD = 10.5). CES-D scores were collected for all 470 participants and 219 were at risk of depression while 251 were considered healthy controls.
Recruitment	Participants were recruited from Amazon Mechanical Turk user base. The only restrictions placed on the sample were age (above 18), nationality (born and raised in the United States), and an "approval rate" (indicating that the participant pays attention and follows instructions correctly in tasks) of over 95%. While all participants are self-selected due to interest and motivation to participate in research studies, this is unlikely to introduce bias into the sample since the participants are more diverse in age, race, and socioeconomic status than typical undergraduate research samples (Buhrmester, Kwang, & Gosling, 2011; Perspectives on Psychological Science), and studies have shown that MTurk workers provide high-quality data that replicates many classic findings in experimental psychology (Piolacci & Chandler, 2014; Current Directions in Psychological Science).
Ethics oversight	The study protocol was approved by Brown University's Institutional Review Board under protocol 1607001555.

Note that full information on the approval of the study protocol must also be provided in the manuscript.
